# National investigation must redefine maternity care in the UK

**DOI:** 10.1016/j.lanepe.2025.101515

**Published:** 2025-11-04

**Authors:** The Lancet Regional Health – Europe

The extent of the crisis in England's maternity services is alarming. Inspections by the Care Quality Commission in 2022–24 rated nearly half of 131 maternity services as requiring improvement or inadequate. Local investigations have reported common problems and repeated mistakes in maternity care across National Health Service (NHS) trusts (ie, organisational units managing hospitals and health-care services), such as failures to listen to women, overlooked safety concerns, poor leadership, and toxic cultures. Concerningly, the UK General Medical Council reported that 27% of obstetrics and gynaecology trainees feel hesitant to escalate concerns about patients to their supervisors—a culture that undermines safety, discourages reporting of errors, and hampers learning.

These problems are well known across the UK, yet meaningful reform is often blocked by complex systems and an overload of local and national recommendations. Chronic underfunding and staff shortages compound the problem: in 2023, England faced a deficit of 2500 midwives, and surveys in 2024 showed widespread concern about unsafe staffing levels across the UK. The financial impact is severe, with £1.3 billion (42%) of 2024–25 clinical negligence payments in England related to maternity.

The human cost is reflected in maternal and newborn outcomes. Maternal mortality in the UK rose to 9.8 deaths per 100,000 maternities in 2020–22, which is the highest since 2003–05, and ranked second-highest in a study of eight high-income European countries, behind only Slovakia and approximately three times higher than in Norway and Denmark. Infant and newborn mortality rates are similarly troubling: in 2022, the UK ranked 19th of 22 comparable Organisation for Economic Co-operation and Development countries. Within the UK, stark socioeconomic and ethnic disparities persist.

A report released in September by MBRRACE-UK, which monitors and investigates UK maternal and newborn deaths, highlighted that women in the most socioeconomically deprived areas are nearly twice as likely to die from pregnancy-related causes as women in the least deprived areas, and Black women are more than twice as likely as White women to die from these causes. These disparities stem not only from the structural determinants of health but also from systemic failures, including a lack of mandatory cultural competency training, flawed data collection, and insufficient accountability from senior leadership. Alarmingly, according to the Health and Social Care Committee, suggestions that ethnic disparities are narrowing reflect how the system is failing all women rather than genuine progress for Black women.

Systemic failures, persistent inequalities, and criminal investigations have eroded trust in NHS maternity care. In response to mounting concerns and public outcry, on June 23, 2025, the UK Government announced a national investigation into maternity and neonatal services in England; the most comprehensive review to date. On Sept 15, 2025, the names of the 14 NHS trusts to undergo scrutiny were announced. Since then, UK maternity services have continued to be the subject of political debate and media coverage. Central to the investigation are safety concerns, a lack of compassionate care, and stark socioeconomic and ethnic disparities. The inquiry aims to produce one comprehensive set of national recommendations for England to drive urgently needed improvements and represents a pivotal moment in beginning to rebuild trust in maternity services.

The national investigation is retrospective, and the main issues identified are likely to be familiar; however, the reports must go beyond listing failures and provide a feasible action plan for improvement. Although unified national recommendations will offer clarity, real change depends on addressing underlying systemic problems. With interim results due next month and the final report in spring 2026, key priorities should include reducing health inequalities; ensuring continuity of carers to foster trust and early problem detection; addressing staff shortages to enable delivery of safe, high-quality care; providing ongoing training in early identification and management of complications; and improving data collection. Most importantly, adequate funding and a blame-free culture where mistakes are openly reported and learnt from—exemplified by Sweden's no-blame compensation scheme—are essential to breaking the cycle of repeated errors.

Failings in maternity care have long plagued the NHS, and countless more women and families will suffer unless a genuine commitment is made to systemic reform across the UK. The true power of this investigation lies in consolidating evidence into an implementable national framework—one that must be matched by political will, sustained investment, and an emphasis on transparency and co-production with the women and families most affected. Only with this support can maternity services take significant steps towards rebuilding trust and ensuring safe, equitable care for all women.

